# Structural and biochemical studies on *Vibrio cholerae* Hsp31 reveals a novel dimeric form and Glutathione-independent Glyoxalase activity

**DOI:** 10.1371/journal.pone.0172629

**Published:** 2017-02-24

**Authors:** Samir Das, Sanghati Roy Chowdhury, Sanjay Dey, Udayaditya Sen

**Affiliations:** 1 Structural Genomics Division, Saha Institute of Nuclear Physics, Kolkata, India; 2 Crystallography and Molecular Biology Division Saha Institute of Nuclear Physics, Kolkata, India; 3 Department of Biotechnology, St. Xavier’s College, Kolkata; Russian Academy of Medical Sciences, RUSSIAN FEDERATION

## Abstract

*Vibrio cholerae* experiences a highly hostile environment at human intestine which triggers the induction of various heat shock genes. The *hchA* gene product of *V*. *cholerae* O395, referred to a hypothetical intracellular protease/amidase *Vc*Hsp31, is one such stress-inducible homodimeric protein. Our current study demonstrates that *Vc*Hsp31 is endowed with molecular chaperone, amidopeptidase and robust methylglyoxalase activities. Through site directed mutagenesis coupled with biochemical assays on *Vc*Hsp31, we have confirmed the role of residues in the vicinity of the active site towards amidopeptidase and methylglyoxalase activities. *Vc*Hsp31 suppresses the aggregation of insulin *in vitro* in a dose dependent manner. Through crystal structures of *Vc*Hsp31 and its mutants, grown at various temperatures, we demonstrate that *Vc*Hsp31 acquires two (Type-I and Type-II) dimeric forms. Type-I dimer is similar to *Ec*Hsp31 where two *Vc*Hsp31 monomers associate in eclipsed manner through several intersubunit hydrogen bonds involving their P-domains. Type-II dimer is a novel dimeric organization, where some of the intersubunit hydrogen bonds are abrogated and each monomer swings out in the opposite directions centering at their P-domains, like twisting of wet cloth. Normal mode analysis (NMA) of Type-I dimer shows similar movement of the individual monomers. Upon swinging, a dimeric surface of ~400Å^2^, mostly hydrophobic in nature, is uncovered which might bind partially unfolded protein substrates. We propose that, in solution, *Vc*Hsp31 remains as an equilibrium mixture of both the dimers. With increase in temperature, transformation to Type-II form having more exposed hydrophobic surface, occurs progressively accounting for the temperature dependent increase of chaperone activity of *Vc*Hsp31.

## Introduction

Different environmental stresses such as elevated temperature and other hostile insults to the living cells accumulate the formation of misfolded proteins and protein-aggregates which may lead to malfunction of the cellular machinery [1]. For the survival of cells, these misfolded proteins need to be either refolded properly into their functionally active form or degraded into their constituent amino acids. Molecular chaperones are a class of folding modulators engaged in quality control of misfolded client proteins and thereby control the cellular conformational ensemble of the proteome [2,3]. Although constitutively expressed under balanced growth conditions, many chaperones are upregulated upon heat shock or other stresses that increase cellular protein misfolding and are, therefore, classified as heat shock proteins (Hsps) [4]. Functionally Hsps are ‘molecular chaperones’ and ‘proteases’ playing central role in cellular homeostasis [2]. They are involved in folding of the hydrophobic stretches of the misfolded client proteins, that normally remain buried but become exposed to the solvent under stressful conditions, through multiple cycles of binding and release. On the other hand, those proteins that are beyond repair are hydrolyzed by the heat shock proteases [5,6]. A typical chaperone target is a short unstructured stretch of hydrophobic amino acids flanked by basic residues and lacking acidic residues [7].

*Escherichia coli* Hsp31 (namely *Ec*Hsp31) (product of *hchA* or *yedU* gene) [6], yeast Hsp31 (YDR533c) [8] and Human DJ-1 [9], belonging to the ThiJ/DJ-1/PfpI superfamily, possess both molecular chaperone and amindopeptidase activities. Members of ThiJ/DJ-1/PfpI superfamily can be subcategorized into three classes based on their structural and functional properties [10]. Due to their multiple cellular functions such as, chaperone-like activity, methylglyoxalase activity and cytoprotection, proteins belonging to this family have emerged as immense biomedical importance [9–12].

*Vibrio cholerae*, the pathogenic organism responsible for diarrhoea, switches from an aquatic environment to a highly hostile milieu on entering the human intestine where it triggers the induction of various heat shock genes to facilitate the breakdown or renaturation of misfolded proteins[13]. The *hchA* gene product of *V*. *cholerae* O395, *Vc*Hsp31, which belongs to DJ-1/PfpI superfamily, is referred as a hypothetical intracellular protease/amidase [14]. However, neither any functional study nor the molecular basis of these functions is reported so far for *Vc*Hsp31.

As of now, our knowledge about the Class-I Hsp31 (*hchA* or *yedU*) is restricted only to the structures of *Ec*Hsp31 [6,15] and its functional assays. *Ec*Hsp31 exists as dimer where each monomer is composed of two α-β domains, designated as A and P domains, connected by a ‘flexible linker’. Dimerization through their P-domains, create a bowl shaped structure. Large hydrophobic patches at the ‘bowl’ may bind partially unfolded protein substrates [6]. The ‘flexible linker’ region and its nearby loops, which were not seen in the crystal form grown at higher temperature, was proposed to function as a gate that modulates high-affinity substrate binding [16]. *Ec*Hsp31 also forms a high molecular weight (HMW) assembly ~50–60°C, with enhanced chaperone activity compared to its dimeric version. However, no atomistic model to precisely describe this mechanism was available [17]. The chaperone activity of *Ec*Hsp31 is also shown to be dependent on the integrity of N- and C-termini [18]. The catalytic triad ‘Cys-His-Asp’ of *Ec*Hsp31 is accessible only through a narrow opening, thereby exhibiting a weak peptidase activity [15,19,20]. *In vitro* protease assay has confirmed poor proteolytic activity towards bovine serum albumin (BSA) with a relatively broad range amidopeptidase activity [16]. *Ec*Hsp31 deficient strains accumulate 8–12 mer long peptide fragments which are cleaved into shorter peptides after incubation with *Ec*Hsp31 [20].

Methylglyoxal (MG; H_3_C—CO—CHO), a reactive carbonyl species, is produced in many organisms as a consequence of central metabolism [21]. MG mediated cytotoxicity and ROS level at elevated concentrations can damage proteins, nucleic acids, and lipids [22,23]. Hsp31 repairs proteins by deglycating cysteine, arginine and lysine and prevents the formation of intermediate Schiff bases and advanced glycation endproducts [24]. It has been demonstrated that *Ec*Hsp31, along with other members of DJ-1 family, exhibit glutathione independent methyl glyoxalase activity and suppresses MG-mediated toxicity and ROS levels [25]. Glyoxalase activities of Hsp31 is also critical for oxidative stress resistance in *Saccharomyces cerevisiae* [26]. However, no protein has been identified in *V*. *cholerae* yet that shows such methylglyoxalase activity.

Here we report high resolution crystal structures of *Vc*Hsp31 and its mutants, grown at three different temperatures ranging from 18°C-25°C, to understand the molecular basis of temperature mediated structural and functional modulations. Our study demonstrates that *Vc*Hsp31 exhibits weak protease/amidase activity, chaperone activity and robust glutathione-independent methylglyoxalase activity. Crystal structures of *Vc*Hsp31 show clear and unambiguous electron density of the ‘linker region’, irrespective of at what temperature the crystals were grown. Quaternary structure analyses, for the first time, indicates the presence of two distinct types of dimers—one is like *Ec*Hsp31 dimer (Type-I dimer) with intersubunit hydrogen bonds involving their P-domains, while in the other (Type-II dimmer) some of the intersubunit hydrogen bonds are broken and the monomers swing out in the opposite directions centering on their P-domains. Crystals of *Vc*Hsp31 grown at 18°C are exclusively made of Type-I dimers whereas crystals grown at 20°C or 25°C have both Type-I and Type-II dimers. Swinging of the monomers uncovers huge buried surface (~400Å^2^), mostly hydrophobic in nature, which might be utilized to bind partially unfolded protein substrates. We propose that, with gradual elevation of temperature, Type-I to Type-II transformation occurs progressively with associated increase of previously hindered hydrophobic surface and this observation reveals a mechanism of temperature dependent increase of chaperone activity. The protease/amidopeptidase activity of *Vc*Hsp31, albeit low, increases with temperature and a double mutant F30L/F211L exhibits two fold greater peptidase activity than the wild type because of increased accessibility of the catalytic cysteine. Through further mutational studies on *Vc*Hsp31 we have deciphered the role of highly conserved residues around the active site in terms of amidopeptidase and methylglyoxalase activities. Our observations further suggest that Glu79 of Hsp31, which is located adjacent to the catalytic Cys and conserved in all three classes, plays a dual role: while it serves as a crucial catalytic residue for methylglyoxalase activity, its strategic location helps to control the proteolysis by attenuating the peptidase activity.

## Materials and methods

### Cloning, expression and purification of VcHsp31 and its mutant

Cloning, expression, purification and crystallization of *Vc*Hsp31 have been published earlier [14]. In brief, *Vc*Hsp31 was cloned in pET28a^+^ vector, overexpressed in *E*.*coli* BL21 (DE3) cells as N-terminus 6×His tagged fusion protein and purified by Ni^2+^-NTA affinity chromatography. The 6×His tag was cleaved with thrombin by overnight incubation at 4°C and the proteins were further purified by gel filtration chromatography using a Sephacryl S-100 (GE-Healthcare) column (78×1.4cm) pre-equilibrated with thrombin cleavage buffer (20 mM Tris-HCl pH 8.0, 150 mM NaCl and 5 mM CaCl_2_) containing 0.02% sodium azide at 20°C. Finally, *Vc*Hsp31 was concentrated upto 10 mg/ml in 20 mM Tris-HCl (pH 8.0), 150 mM NaCl and 2.5 mM CaCl_2_. To generate different *Vc*Hsp31-Ala and Leu mutants, two step PCR methods were applied to introduce specific mutations into *Vc*Hsp31-pET28a^+^ plasmid and confirmed by sequencing.

### Crystallization and data collection of *Vc*Hsp31

Crystals of *Vc*Hsp31 and its mutants were grown by hanging drop vapor diffusion method at different temperatures, from 18°C-25°C, in presence of 5% PEG 6000 in 0.1 M Na-citrate (pH 5.0) and 8% 2-methyl-1,3-propanediol (MPD) against a reservoir solution of 15% PEG 6000, 0.1 M Tris—HCl (pH 8.5) and 5% MPD. *Vc*Hsp31 crystals grown at 20°C diffract to a resolution of 1.9 Å whereas crystals grown at 25°C diffract to a resolution of 2.5Å. Crystals of E79A-*Vc*Hsp31 (at 18°C) and C188A-*Vc*Hsp31 (at 20°C) mutants were also grown in a condition similar to that of wild type *Vc*Hsp31 and diffract to a resolution of 2.3 Å and 1.85 Å, respectively. Attempts to crystallize *Vc*Hsp31 at a temperature higher than 25°C either produced no crystals or crystals with no suitable X-ray diffraction.

For data collection, crystals were taken out from the crystallization drops using nylon loop, briefly soaked in cryoprotectant solution and flash-cooled in a stream of nitrogen (Oxford Cryosystems) at 100K. The diffraction data sets were collected using a MAR Research image-plate detector of diameter 345 mm and Cu K_α_ radiation generated by a Bruker—Nonius FR591 rotating-anode generator equipped with Osmic Max Flux confocal optics and operated at 50kV and 70mA. Data were processed and scaled using AUTOMAR (http://www.marresearch.com/automar/run.html). Data-collection and processing statistics are given in Table 1.

**Table 1:** Data collection and refinement statistics of VcHsp31 and its different mutants.

	VcHsp31^20°C^	VcHsp31^25°C^	E79A-VcHsp31 (18°C)	C188A- VcHsp31(20°C)
***Crystal cell parameters***				
Space group	*P*2_*1*_	*P*2_*1*_	*P*2_*1*_	*P*2_*1*_
Cell dimensions (Å, °)	a = 73.5,b = 79.1,c = 133.1; α = γ = 90.0, β = 95.2	a = 73.3,b = 80.0,c = 133.1; α = γ = 90.0, β = 95.4	a = 104.4,b = 79.6,c = 106.4; α = γ = 90.0, β = 107.9	a = 103.7,b = 79.3c = 107.6; α = γ = 90.0, β = 108.7
***Data collection statistics***				
Molecule(s)/ASU	6	6	6	6
Mathews coefficient, V_m_ (Å^3^ Da^-1^)	1.97	2.04	2.12	2.11
No. of observed reflections	206188	94575	132035	320035
No. of unique reflections	118593	49277	68904	130789
Resolution range (Å)	30.0–1.87	30.0–2.5	30.0–2.3	30.0–1.84
Completeness (%)	92.0 (92.3) ^a^	92.3 (87.6) ^a^	92.2 (95.0) ^a^	91.1 (95.0) ^a^
Average redundancies	1.79	1.94	2.1	2.5
Mosaicity	0.36	0.43	0.44	0.38
R_merge_† (%)	8.6 (34.2) ^a^	5.7 (25.6) ^a^	5.7 (43.2) ^a^	5.2 (46.3) ^a^
Average I/σ	8.9 (21.1) ^a^	8.9 (3.3) ^a^	6.7 (1.5) ^a^	7.3 (1.6) ^a^
***Refinement statistics***				
No. of reflections used	118593	49277	68904	130789
R_free_ (%) ^c^	19.6	25.7	24.6	23.8
R_cryst_ (%)^b^	15.8	17.8	17.9	18.9
r.m.s.d bond (Å)	0.01	0.008	0.008	0.006
r.m.s.d bond (°)	1.35	1.2	1.2	1.07
***Ramachandran plot (%)***				
Most favored(%)	90.1	95.1	93.9	96.2
Allowed(%)	8.5	4.8	5.6	3.6
Disallowed(%)	0.4	0.1	0.5	0.1

^a^ Numbers in parentheses are values for the highest-resolution bin.

^†^R_merge_ = ∑hkl∑i׀Ihkl-〈Ihkl〉׀/∑hkl∑i(Ihkl), where Ihkl is the intensity of an individual reflection and 〈Ihkl〉 is the average intensity over symmetry equivalents

^b^ R_cryst_ = ∑hkl׀F_obs_-F_calcd_׀/∑hklF_obs_, where F_obs_ and F_calcd_ are the observed and calculated structure factor amplitudes, respectively.

^c^ R_free_ is the equivalent of R-factor, calculated for a randomly chosen set of the reflections (5%) that were omitted throughout the refinement process.

^d^ As defined by PROCHECK.

### Structure determination and refinement

The structures were solved by molecular replacement using *Phaser* [27] and refined in *Phenix* [28]. At first, we solved the structure of *Vc*Hsp31 with the high resolution data collected at 20°C (*Vc*Hsp31^20C^). Based upon sequence alignment between *Vc*Hsp31 and *Ec*Hsp31, a model was generated from the coordinates of the *Ec*Hsp31 (1N57.pdb) [6], by the program *Chainsaw* from the CCP4 package [29]. This model was used to generate the initial solution by molecular replacement using *Phaser* [27]. The solution was found to contain six molecules in the asymmetric unit which correspond to a Matthew's coefficient V_M_ of 1.97 Å^3^ Da^−1^ with a solvent content of 37.4%. Cycles of model building in coot [30] and refinement by *Phenix* resulted in final R_work_ and R_free_ values to 18.8% and 22.4%, respectively. This refined structure was subsequently used to solve the *Vc*Hsp31^25C^ and the mutant structures E79A-*Vc*Hsp31and C188A-*Vc*Hsp31. In each case, 5% of the reflections were kept aside for cross validation prior to refinements.

### Structural analysis

Average B-factors for each residue was calculated using B-avg of CCP4. PISA web server was used to analyze the oligomeric states [31]. Sequence comparison of the protein with other homologs was done using ClustalW [32] and Multalin software [33]. All the structural illustrations were prepared using pymol (www.pymol.org).

### Normal mode analysis

The low frequency normal modes were used to model the conformational flexibility of the monomers using the web-server elNémo [34]. The A:B dimer (Type-I) of the *Vc*Hsp31 crystal structure was used as the initial model. A series of low frequency normal modes were calculated and perturbations were applied to the lowest vibrational mode (mode 7) with amplitude varying from -100 to +100 in steps of 20. A total of 11 models were generated by the program.

### Tryptophan quenching study

2 μg of VcHsp31 was added in 20 mM Tris, 150 mM NaCl, pH-8.0 making the final volume 1200 μl and incubated at the temperature range from 18°C to 30°C in Varian-Cary fluorescence spectrophotometer with the temperature control unit or peltier. The protein sample was excited at 295 nm and emission spectra were collected from 320 nm to 450 nm.

### Protease and peptidase assay

Proteolytic activity of *Vc*Hsp31 was conducted at two different temperatures (20°C and 37°C) and in three different pHs (6.5, 7.2, and 8.0) using BSA (Sigma Aldrich) as substrate. The assay buffer at pH 8.0 contained 150 mM NaCl, 2.5 mM CaCl_2_, 10 mM dithiothreitol and 20 mM Tris. For the assay at pH 6.5 and pH 7.2, MES and phosphate buffer was used to a final concentration of 20 mM, respectively, while all other components were kept unaltered. The ratio of *Vc*Hsp31 and BSA was kept to 5:1 to account for the weak proteolytic activity of *Vc*Hsp31. The proteolytic reactions were monitored for defined periods (15, 24, 40 hrs) and after that the reactions were stopped by the addition of 1% SDS in final mixture. The proteolyses products were analyzed by SDS-PAGE.

Peptidase activity of *Vc*Hsp31 was determined by monitoring the production of Amino Methyl Coumarin (AMC) from the fluorogenic peptide Ala-AMC (Sigma Aldrich). 2 μg of *Vc*Hsp31 was incubated for 12 hrs with 300 μM substrate in 20 mM Tris (pH 8.0), 1 mM dithiothreitol and 3% Dimethyl sulfoxide in a total volume of 100 μl. AMC fluorescence (E_x_λ 380 nm and E_m_λ 488 nm) was measured after the reaction was stopped by the addition of 1% SDS in final mixture. Each rate was measured in triplicate and mean values are plotted in originpro 8 with standard deviations (calculated in Microsoft excel) shown as error bars. Temperature dependency of the peptidase activity was assayed at seven different temperatures: 20°C, 25°C, 30°C, 35°C, 37°C, 39°C and 42°C. Effects of metal ions on the peptidase activity of *Vc*Hsp31 were assayed in the presence of five different divalent cations: Mg^2+^, Ca^2+^, Zn^2+^, Hg^2+^, and Pb^2+^ keeping the concentration of these metal ions fixed (5 mM).

### Methylglyoxalase activity assay

Glyoxalase activity was assayed in vitro by using a previously described colorimetric method that determines the enzymatic depletion of the substrate methylglyoxal [21]. 10 ng of purified *Vc*Hsp31 was added in the reaction buffer (100 mM sodium phosphate, pH 7.5 containing 1 mM of DDT and 1 mM EDTA) with increasing concentration of methylglyoxal (0.1 mM to 2.5 mM) to make a final volume of 100 μl and incubated at 37°C for predetermined length of time. The reaction was stopped by adding 0.1% 2,4-dinitrophenylhydrazine (DNPH) solution (in 2N HCl) followed by the addition of 210 μl of ddH_2_O. The whole mixture was incubated for 15 minute at room temperature and finally 10% NaOH solution was added. The residual methylglyoxal was determined by reaction with DNPH to generate the characteristic purple color by the chromophore methylglyoxal-bis-2,4-dinitrophenylhydrazone after alkali treatment followed by measurement of absorbance at 550 nm in a spectrophotometer [35,36]. The measured velocities of the reaction were used to plot the Michaelis-Menten equation and to obtain the K_m_ of the reaction. Alanine scanning mutations of catalytic triad residues Cys188 and His189 but not Asp216 (as the protein precipitates) along with some other important residues around the catalytic site (Glu79, His158, Tyr224, His76) were tested to determine their role in glyoxalase activity and compared it with the wild type. The measured absorbance values at 550 nm were converted to concentration of methylglyoxal using interpolation onto the best-fit line for a methylglyoxal-DNPH calibration curve.

### Chaperone activity aggregation suppression assays

In this assays insulin was taken in a buffer containing 20 mM Tris, 150 mM NaCl at pH-7.0 and the aggregation reaction was induced by the addition of Dithiothreitol (final conc. 25 mM) in a 1500 μl cuvette at room temperature. Turbidity resulting from the aggregation of insulin was monitored at 360 nm by right angle scattering method in a Varian-Cary fluorescence spectrophotometer [37]. Increasing concentration of *Vc*Hsp31 (substrate: enzyme ratio was 15:1, 3:1, 1:1, 1:6) were used to check the dose dependency of chaperone activity. Data were plotted using Microsoft EXCEL and originpro 8.

## Results

### Structure of *Vc*Hsp31 and its comparison with other Hsp31 orthologs

The asymmetric unit of *Vc*Hsp31^20C^, *Vc*Hsp31^25C^, E79A-*Vc*Hsp31 and C188A-*Vc*Hsp31 crystals contains six Hsp31 molecules (chain A to F) and all the six chains have continuous electron density except for the four N-terminal residues and the C-terminus Lys residue. Graphical inspection of the packing and its analysis by the program PISA [31] indicate that six monomers assemble into three dimers. The structures of *Vc*Hsp31^20C^, *Vc*Hsp31^25C^, E79A-*Vc*Hsp31 and C188A-*Vc*Hsp31 refined well with R_work_/R_free_ values of 15.8/19.6%, 17.8/25.7%, 17.9/24.6% and 18.9/22.3% respectively, and excellent stereochemistry as indicated from their Ramachandran plots (Table 1) [38]. The monomers superpose well with r.m.s.d values ranging between 0.15 to 0.25 Å for 280 Cα atoms, implying all of *Vc*Hsp31 chains acquire identical overall structures.

**Fig 1:**
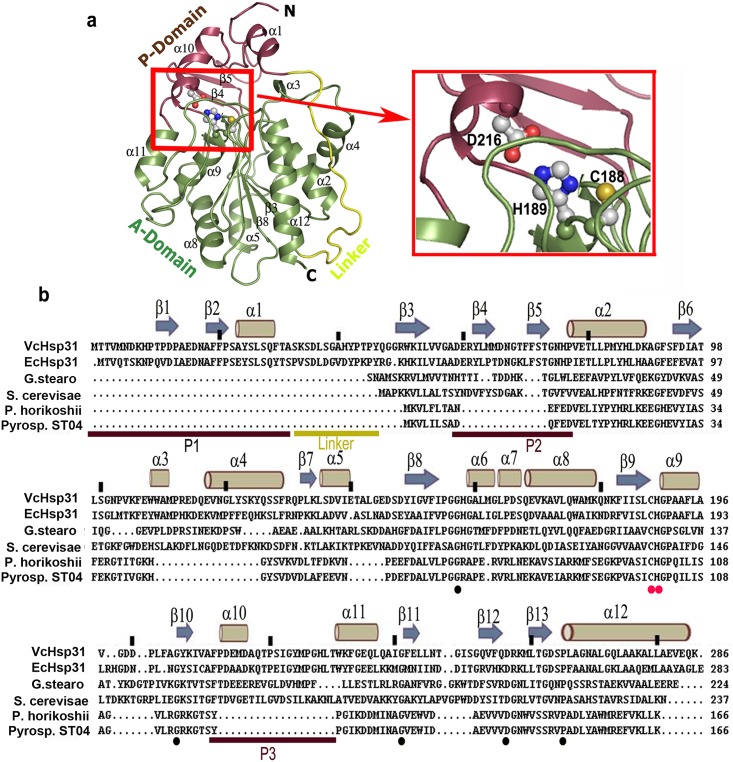
Cartoon representation and sequence alignment of *Vc*Hsp31 with other orthologs showing domain organisation and catalytic residues. **(a)** Cartoon representation of the overall structure of *Vc*Hsp31 monomer where ‘A’ domain (green), ‘P’ domain (violet) and the ‘linker’ region (yellow) are indicated. The catalytic triad region comprising Cys188-His189-Asp216 (small red box) is zoomed for clarity; **(b)** Structure based sequence alignment of the three classes of Hsps taking two representative members from each class. Top of the alignment depicts their secondary structures and every twentieth residue of *Vc*Hsp31 is marked by a (|). At the bottom of the alignments, P domain segments and ‘linker region’ are indicated by colored bars. Catalytic residues are marked in red dots while residues conserved in all six proteins are in black dots.

*Vc*Hsp31 monomers have a structural organization of two α-β domains, identified as A and P domains (Fig 1a and 1b). The larger ‘A-domain’ has a α/β hydrolase fold consisting of 201 amino acids with a central β-sheet (consisting of six parallel strands β7, β6, β3, β8, β9 and β13 together with solitary antiparallel strand β12) flanked by helices α2, α3, α4, and α11 on one side and helices α5, α6, α7, α8, and α10 at the other (Fig 1a and 1b). The shorter P-domain (68 residues), which acts as a lid on the top of A-domain, is composed of three segments: P1 segment (Met1-Ala32), P2 segment (Asp59-Asn75) and P3 segment (Pro212-Thr230) (Fig 1a and 1b). The secondary structure of P-domain is composed of an antiparallel β-sheet (β1↑-β2↓- β5↓- β4↑) flanked by two helices, α1 and α10. The catalytic triad residues Cys188 and His189 are contributed by the A-domain while Asp216 is contributed by the P-domain (Fig 1a). The ‘linker region’ (Fig 1a and 1b) tethers the P and A domains through a number of electrostatic interactions.

**Fig 2:**
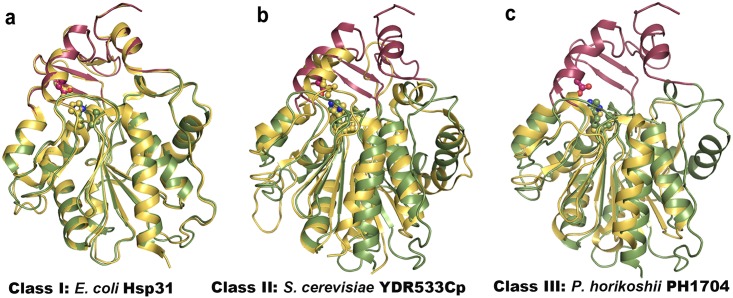
Superpositions of VcHsp31 with three different Hsp31 orthologs. **(a)** EcHsp31 (Class-I) [PDB ID 1PV2, sequence identity 58.885% (calculated in ClustalO), rmsd 0.428] [15] **(b)** YDR533Cp from *Sachharomyces cerevisiae* (Class-II) [PDB ID 4QYX, sequence identity 18.033%, rmsd 2.932] [41] and **(c)** PH1704 from *Pyrococcus horikoshii* (Class-III) [PDB ID 1G2I, sequence identity 10.69%, rmsd 1.533] [40] Hsps to show their domain organization. VcHsp31 is shown in same color as in Fig 1a).

Both *Vc*Hsp31 and *Ec*Hsp31 belong to the Class-I family of Hsp31 due to the presence of a complete P-domain, involved in dimer formation and the presence of an extended ‘linker region’ (Figs 1a and 2a). Monomers of *Vc*Hsp31 share similar structure with *Ec*Hsp31 (Fig 2a) as they superpose with an r.m.s.d of 0.43Å for 236 atoms. Class-II orthologs (such as YDR533c from *Sachharomyces cerevisiae* and APC35852 from *Geobacillus stearothermophilus*) have an incomplete P-domain and no ‘linker region’[8]. Due to the incomplete P-domain the catalytic Cys is more accessible in case of the Class-II orthologs (Fig 2b). A Glu residue, instead of an Asp, completes the catalytic triad (Figs 1b and 2b). Class-III orthologs PH1704, a protease from *Pyrococcus horikoshii* [39], I3RG07, an intracellular protease from *Pyrococcus sp*. *ST04* and and PfpI [40], an intracellular protease from the hyperthermophilic *Sulfolobus solfataricus* are devoid of P-domain and the ‘linker region’ (Fig 2c). A Glu residue from the adjacent protomer completes the catalytic triad. Although the overall fold of their A-domains match well, the helical region corresponding to α3-α4 of *Vc*Hsp31 adopt a β-strand conformation in Class-III orthologs (Fig 2c).

### Dimeric structure of *Vc*Hsp31

**Fig 3:**
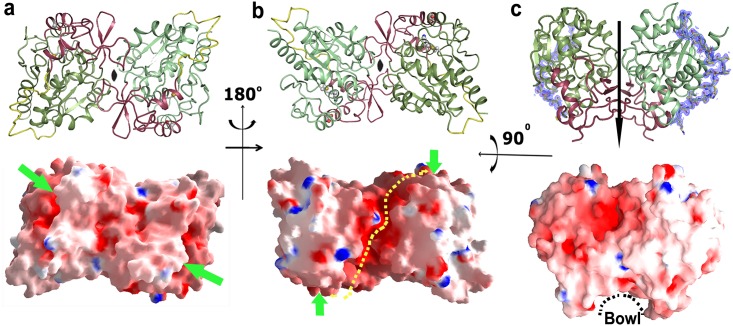
Cartoon representation of *Vc*Hsp31 dimer in different orientations and their electrostatic surface (a-b) looking down the twofold symmetry and (c) perpendicular the twofold. Electron density contoured at 1σ, overlaid on the linker region (in sticks), is shown in blue mesh (Top). Electrostatic potential surface of *Vc*Hsp31 dimer shown with an orientation same as in cartoon representation. Canyon (yellow dots), bowl and the location of the catalytic triad (green arrow) are indicated (bottom). Color code used is same as Fig 1a, a lighter shade is used for the other monomer.

Structures of *Vc*Hsp31^20C^, *Vc*Hsp31^25C^, *Vc*Hsp31-E79A and *Vc*Hsp31-C188A contain six monomers per asymmetric unit and analysis of the buried surface area between monomers indicates that they assemble into three homo-dimers where the monomers are related by a twofold axis (Fig 3c). Dimerization of *Vc*Hsp31 burry about 2050Å^2^ surface area (Table 2) which involves interactions between P-A´ domains (‘´’ indicates the domain from the other monomer) and P-P´ domains. Fig 3a–3c shows the dimer in three different orientations along with their surface potential. It is evident from Fig 3a and 3b that the majority of the surface of *Vc*Hsp31 is uncharged in nature except for the region at the waist of the dimer. At the waist region, dimerization of *Vc*Hsp31 creates a deep canyon (Fig 3b) with a 8–14 Å gap between monomers. The canyon is highly negative in charge and extends towards the other face of the molecule connecting two other negatively charged grooves that harbors the two active sites. Negative charge of the canyon is formed by the clustering of acidic residues Asp16, Asp17, Asp59, Glu60, Asp66, Asp95, Asp137, Glu140, Glu166, and Glu169 from each monomer. A large shallow hydrophobic surface is evident from Fig 3c which is about 20 Å in diameter and contributed mainly by the residues from the P-domain.

**Table 2:** Interface area (Å^2^) and dimer types for different *Vc*Hsp31 crystals.

Dimers	*Vc*Hsp31^20°C^	*Vc*Hsp31^25°C^	E79A-*Vc*Hsp31(18°C)	C188A-Hsp31(20°C)
A-B	2025 (Type-I)	2058 (Type-I)	2104 (Type-I)	2100 (Type-I)
C-D	2063 (Type-I)	2082 (Type-I)	2042 (Type-I)	2084 (Type-I)
E-F	1704 (Type-II)	1663 (Type-II)	2034 (Type-I)	1724 (Type-II)

**Fig 4:**
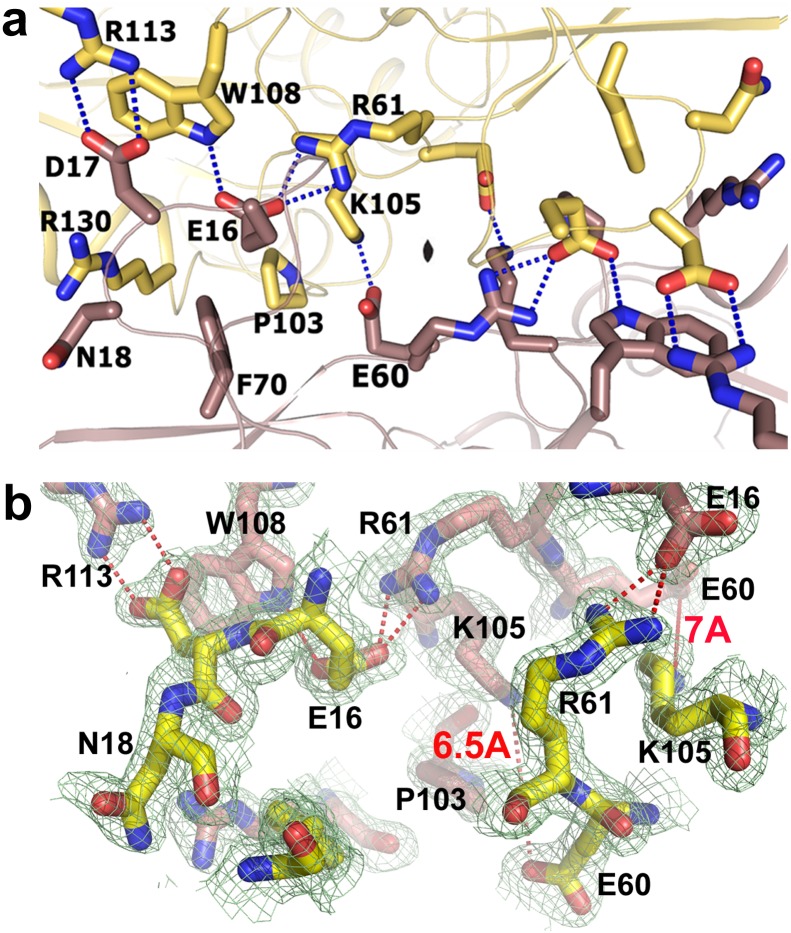
Interactions at the dimeric interface in Type-I and Type-II dimers. (a) Interactions at the A-B and C-D dimeric interface of *Vc*Hsp31. Two monomers are shown in yellow and brown and strong electrostatic interactions are shown in dashed line. (b) Electron density contoured at 1σ at the E-F dimeric interface showing that the salt bridge between K105 and E60 is abolished here (red label).

Dimeric structure of *Vc*Hsp31 is evident in solution (S1 Fig) and is stabilized through several salt bridges and hydrophobic interactions at their interfaces. These interactions are depicted in Fig 4a and 4b and tabulated in Table 3. At the interface, conserved salt bridges E60…K105', E16…R61' and D17…R113' along with several strong hydrogen bonds (Table 3) and hydrophobic packing of F70 with P103' stabilize the dimeric structure. Because of two-fold symmetry, these interactions are occurring twice providing a net free energy of interaction of -5 kcal/mole, as calculated by *PDBePISA* webserver. Overall, the residues and the interactions at the dimeric interface are similar in *Vc*Hsp31 and *Ec*Hsp31, implying that this kind of dimerization is conserved in Class-I Hsp31 homologs and probably essential for their function.

**Table 3:** Interactions at the interface of A-B and E-F dimer.

Residues	Residues	Distance (Å)	
		AB	EF
Ala 15 O	Arg 61 NE1	2.9/3	3.6/3.6
	Arg 61 NH1	2.9/2.9	3.5/3.55
	Arg 61 NH2	2.9/2.8	3.3/3.2
Glu 16 OE1	Trp 108 NE1	2.9 /3	2.9/2.95
Glu 16 OE2	Arg 61 NH1	2.9/2.9	2.7/2.8
Glu 16 OE2	Arg 61 NH2	2.9/2.8	3.0/2.9
Asp 17 OD1	Arg 113 NH2	3/3	2.9/3.0
Asp 17 OD2	Arg 113 NH1	2.8/2.8	3.3
Asp 59 OD1	Asn 102 ND2	3.2/3.3	3.5/3.7
Asp 59 OD1	Asn 102 ND2		3.5/3.2
Glu 60 OE1	Lys 105 NZ	2.8/2.6	
Glu 60 OE2	Lys 105 NZ	3.5 /3.5	

### Two distinct dimers in the same asymmetric unit of *Vc*Hsp31

**Fig 5:**
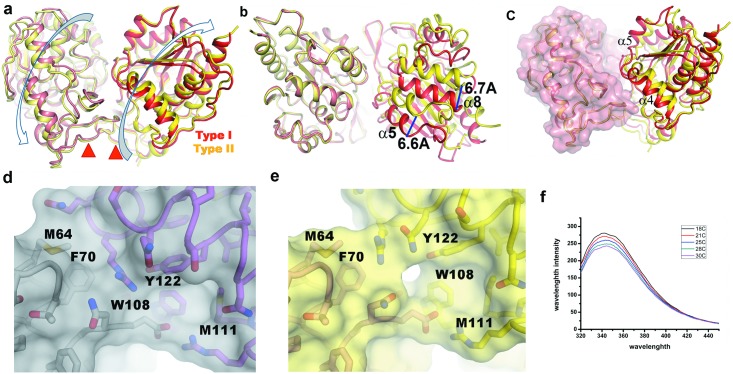
Type-I and Type-II dimer of *Vc*Hsp31. (a) Overall superposition of the dimers (viewing perpendicular to the bowl). Direction of swinging motion required to form Type-II dimer from Type-I dimer is shown by the flat arrow and the position of pivot point is shown in red triangle; (b) Disposition of ‘chain B’ and ‘chain F’ (viewing from top of the canyon) when ‘chain A’ and ‘chain E’ are superposed. Large displacements of helices are evident here; (c) Same superposition scheme as in Fig 5b but chain A is shown as surface (viewing same as Fig 5a) which shows poor packing of α4 for Type-II dimer with chain E (shown in surface) (d) A portion of buried dimeric surface in Type-I dimer which is being exposed (e) in Type-II dimers; (f) Tryptophan quenching of *Vc*Hsp31at low temperatures (18°-25°C).

Crystal structures of *Vc*Hsp31 or its mutants contain three dimers in the same asymmetric unit and analysis of their structures shows that except *Vc*Hsp31-E79A (grown at 18°C) all of them contain two types of dimers. Presence of two dimeric species was not evident when six monomers were superposed (r.m.s.d. values 0.15 Å to 0.25 Å for 278 Cα atoms), indicating they all of them possess very similar overall fold. The scenario is, however, different when superposition was done between the dimers. For *Vc*Hsp31^20C^, A-B dimer superposes on E-F dimer with an r.m.s.d of 1.80 Å (for 561 Cα atoms) whereas the corresponding value is only 0.22 Å with the C-D dimer (Table 4). This implies that A-B and C-D dimers (named as Type-I dimer) are considerably different from E-F dimer, which is Type-II dimer, with respect to the relative orientations of the constituting monomers (Fig 5a). Two types of dimer with similar features have also been observed for *Vc*Hsp31^25C^ and C188A-*Vc*Hsp31. Comparison of the interactions at the dimeric interfaces of Type-I dimer with Type-II shows that several interactions at the dimeric surface are lost or weakened in the latter (Table 3). As a result, monomers swing out in the opposite directions of about 8° around their respective P-domains as pivot point, like twisting of wet cloth, forming a novel dimeric organization (Fig 5a). The extent of swing is more evident when chain-A of A-B dimer is superposed on chain-E of E-F dimer and the relative orientations of B and F are compared. Fig 5b shows such a superposition from the top of the canyon, where some of the helices are found to be displaced by more than 6 Å. Looking along the bowl (Fig 5c), where chain A is shown as transparent surface, region α4/β7/α5 of chain B is seen to pack with chain A and gets buried during dimer formation. However, in E-F dimer corresponding part of chain F is no longer in a position to pack with chain E and these regions remain partially exposed to the solvent. Thus, swinging of the monomers provide a mechanism to expose huge buried surface (~400Å^2^) at the dimeric interface, mostly hydrophobic in nature, which might be utilized to bind partially unfolded protein substrates (Table 2). There are two tryptophan residues (Trp108, Trp109) from each monomer residing near the bowl which are partially exposed in E-F dimer at higher temperature. As small rise in temperature (18°C to 25°C) is not enough to cause detectable conformational change, it should not affect Trp quenching as well. With this assumption we collected the Trp quenching data of *Vc*Hsp31 from 18°C to 30°C which shows a red shift and gradual decrease of intensity, typically seen when Trp is getting exposed, indicating that the kind of swinging motion seen in the crystal structure is also occurring in solution (Fig 5d).

**Table 4:** RMSD values obtained by dimer-dimer superposition.

Dimer	*Vc*Hsp31^20°C^	*Vc*Hsp31^25°C^
A-B vs E-F	1.74	1.89
C-D vs E-F	1.72	1.79
A-B vs C-D	0.21	0.31

**Fig 6:**
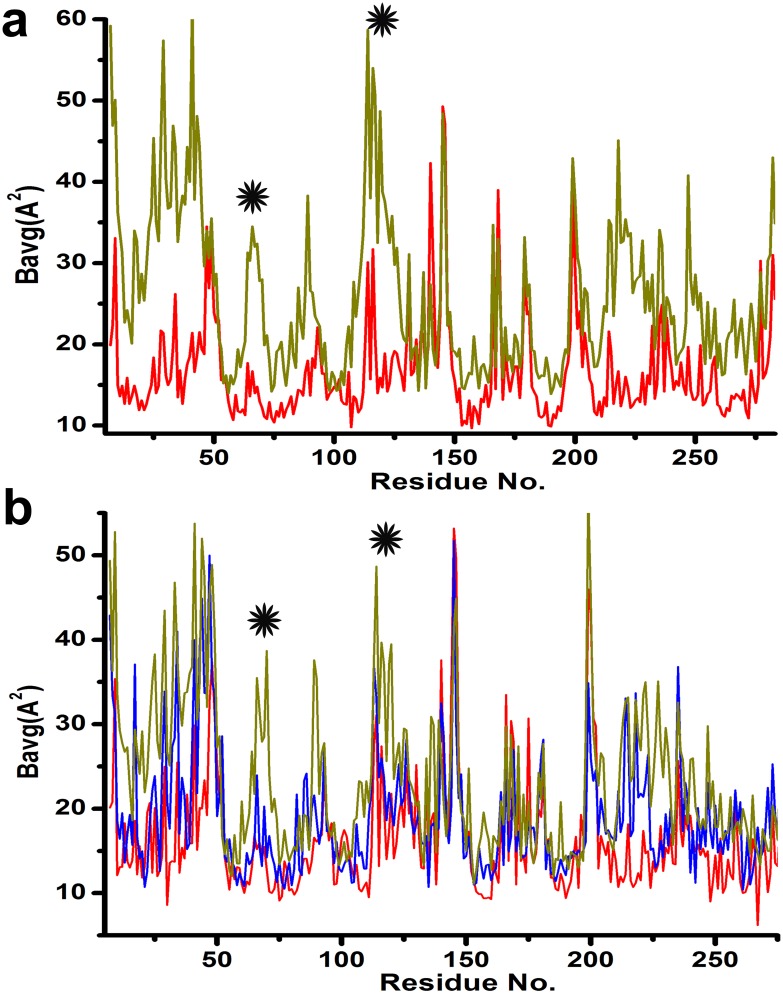
B-factor plot of different dimers. (a) Temperature factor plot of Type-I dimers of *Vc*Hsp31^20C^, B averages of chain A (red) and chain E (green) are plotted as representative (b) B averages of Type-II dimers of *Vc*Hsp31^25C^ are plotted with chains A (red), C (blue), E (green) as representative. Regions α4/β7/α5 and β4/β5 loop, that loose contact at the dimeric surface upon swinging motion in type-II dimer are shown by asterisk.

Type-I and Type-II dimers also differ in their thermal parameters. Overall B-factor of the monomers of *Vc*Hsp31 (at 20°C and 25°C) and a graphical representation of the B-average of their different chains are shown in Table 5 and Fig 6a and 6b. Since the six monomers belong to the same asymmetric unit, their B factors were compared without any normalization. Moreover, as all the crystals of *Vc*Hsp31 were grown using same precipitants and were flash frozen in a similar way, normalization were not done between them either. In case of *Vc*Hsp31^20C^ structure, F and E chain has high average B-values whereas the B-values for A, B, C and D chains are low and comparable, (Fig 6a, Table 5). Interestingly, B-values for α4/β7/α5 and β4/β5 loop in Type-II dimer (E-F chain) are higher (Fig 6a) as these regions loose contacts with its partner due to swinging motion. For *Vc*Hsp31^25C^ structure, average B-values of A, B chains and E, F chains are quite comparable to that of *Vc*Hsp31^20C^ while C, D chains have intermediate average B-values Fig 6b. We presume that increase of temperature (from 20 to 25) some Type-I (C-D) dimer have just started transforming towards type-II dimer. Further increase of temperature would completely transform these dimers (C-D) and at that temperature there would be 66% of type-II dimer.

**Table 5:** Average B-factors of different chains of *Vc*Hsp31.

Chains	B-average *Vc*Hsp31^20°C^	B-average *Vc*Hsp31^25°C^
Chain A	17.8	17.8
Chain B	17.1	17.3
Chain C	19.0	21.2
Chain D	18.4	20.1
Chain E	24.2	24.1
Chain F	26.4	26.6

### The catalytic triad and its environment

The catalytic triad ‘Cys188-His189-Asp216’ of *Vc*Hsp31 is located at the junction of the P and the A domains where A-domain harbors Cys188 and His189 while P-domain harbors Asp216 (Fig 1a). The catalytic Cys188 is encompassed within a nucleophile elbow-like motif [42] and is located at the tip of a tight turn between β9-α9 (Fig 1a and 1b). The ϕ/ψ (~60°/~70°) values of this residue fall in the unfavourable region of the Ramachandran plot indicating that this residue bears an unusually strained condition. The same conformational feature of the catalytic Cys has been observed in the structures of DJ-1, *Ec*Hsp31, and PH1704. The constellation of the catalytic triad, as seen in *Vc*Hsp31 is similar to that of *Ec*Hsp31, PH1704 and papain-type cysteine proteases where the sulfhydryl group of Cys188 interacts with the ND1 of His189 and the Asp216 interacts with the NE2 of His189 (~2.7 Å). In addition, Glu79 which is analogous to Glu15 of PH1704, is also seen to make hydrogen bond with the sulfhydryl group of Cys188. Strong electrostatic interactions are also observed between Glu107 OE1 with His76 ND1 (~3.3 Å) and His158 NE2 with Asp216 OD1 (~3.0 Å) near the active site. The imidazole ring of His158 and the main chain NH of His158 and Gly157 together fulfill the role of an ‘oxyanion hole’ that could stabilize the transition state for efficient hydrolysis. The catalytic triad Cys188-His189-Asp216 and the residues His76, Glu79, Glu107, Gly157, His158 and Tyr224 are highly conserved in Class-I Hsp31s. In case of Class-II Hsp31 homologues active site aspartate is substituted by a glutamate and residues corresponding to His76, Tyr224 and Met225 are replaced by aromatic and small hydrophobic residues (Fig 1b). In Class-III Hsp31 homologues, except for the catalytic triad residues, all other residues are different. Interestingly, residue that corresponds to Glu79 of Class-I Hsp31s, is found to be conserved in all Hsp31s (Fig 1b). Being devoid of P-domain, Class-III Hsp31 homologues completes the catalytic triad through a Glu74´ residue which is contributed by its dimeric partner.

### Accessibility of the catalytic triad

**Fig 7:**
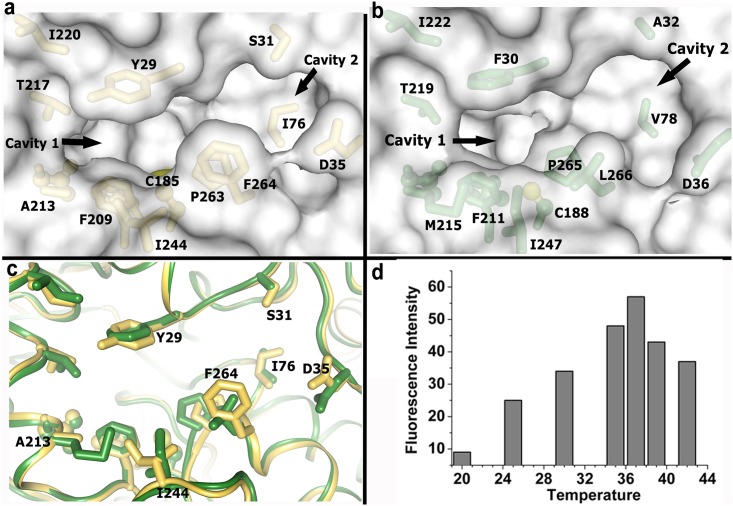
Access of catalytic triad through two cavity pocket. (a) Residues involved in forming the two cavity pocket in *Ec*Hsp31. Cavity 1 and Cavity 2 are indicated by arrow; (b) Two cavity pocket in *Vc*Hsp31 in the same orientation to that of *Ec*Hsp31; (c) Superposition of residues forming the two cavity pocket in *Ec*Hsp31 and *Vc*Hsp31. Residues of *Vc*Hsp31 that differ in sequence or having structural alterations with *Ec*Hsp31 are only labeled; (d) Temperature dependence of amidopeptidase activity of *Vc*Hsp31 determined using Ala-AMC as substrate.

For *Ec*Hsp31, the catalytic Cys residue is accessed through a two cavity pocket (Fig 7a). The boundary of the ‘cavity 1’, through which the catalytic Cys is accessed, is defined by two layers of residues. The layer immediate to Cys is formed by Pro263, Phe209 and His155 and the layer on top of it that face the surface is formed by Tyr29, Ala213, Thr217, Ile244 and Phe264 (Fig 7a). The catalytic Cys is accessed through these layers with a narrow channel of only 6Å radius opening (Fig 7a). If we compare this region of *Vc*Hsp31 with the *Ec*Hsp31 we see several changes either in terms of amino acids substitution or altered side chain conformations (Fig 7b). Although the residues in the first layer are conserved and have similar spatial disposition in *Vc*Hsp31, the layer on top of it has a number of substitutions/structural alterations (Figs 7b and 1b) which effectively opens up the mouth of the cavity in *Vc*Hsp31 (Figs 7c and 1b). In this case, the larger size of Met215 (compared to Ala213 in *Ec*Hsp31) pushes the side chain of Ile244 and the later takes a conformation different from that of *Ec*Hsp31 (Fig 7b and 7a). As a result, the larger size of Met215 is compensated by the altered conformation of Ile244 side chain as far as that part of ‘cavity 1’ is concerned (Fig 7c). However, residues like Phe30 and Leu266 of *Vc*Hsp31, whose side chains are smaller in sizes than Tyr29 and Phe264 of *Ec*Hsp31 respectively, opens up the ‘cavity 1’ (Fig 7b and 7c) of *Vc*Hsp31 a little (~0.3 Å longitudinally) but more importantly ‘cavity 1’ gets connected with the ‘cavity 2’. In *Ec*Hsp31 ‘cavity 2’ is shallow and is connected to ‘cavity 1’ through a narrow passageway between Pro263 and His74 which is only ~5Å wide. In *Vc*Hsp31, shorter side chains Ala32, Val78, Leu266 of *Vc*Hsp31 compared to Ser31, Ile76 and Phe264 of *Ec*Hsp31 and altered conformation of Asp36 (Asp35 in *Ec*Hsp31) make ‘cavity 2’ bigger (approx. 11.5 Å diameter in VcHsp31 compared to 8.2 Å diameter in EcHsp31 in vertical direction) and connected to the ‘cavity 1’ through a broader passageway (Fig 7a and 7b).

### Protease and amidopeptidase activity of *Vc*Hsp31 at different temperatures

**Fig 8:**
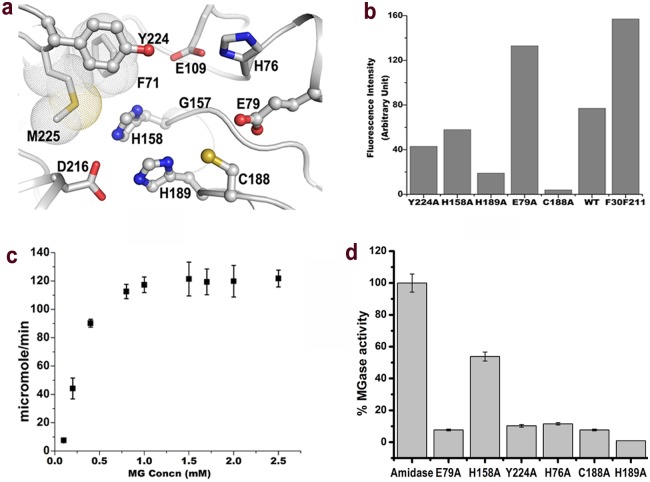
Influence of highly conserved residues around catalytic site on peptidase and methylglyoxalase activity. (a) Amino acid residues around the catalytic C188 of VcHsp31 which are mutated for functional studies. Among them residues in ball-and-stick are those for which functional studies have been done. Residues in stick were mutated but the resulting protein has poor solubility and eventually not included for functional studies. Residues Met225 and Phe71 are shown in dot-surface. (b) Peptidase activity of different mutants measured using Ala-AMC as substrate at 37°C. (c) Methylglyoxalase activity of *Vc*Hsp31 plotted against substrate concentration. (d) Methylglyoxalase activity of different *Vc*Hsp31 mutants.

Unlike other Cys proteases present in mammalian lysosomes, the protease activity of VcHsp31 is very low at 20°C and in acidic pH (~6.5). However, about 50% of BSA is cleaved, at pH 8.0 and 40 hrs of incubation at 20°C while at 37°C almost 100% cleavage was observed (almost two times faster) within ~24 hrs of incubation (S2 Fig). The amidopeptidase activity of *Vc*Hsp31 has also been studied using Ala-AMC as substrate where a gradual increment of AMC concentration is found upto 12 hrs of incubation at 37°C (S2 Fig). Amidopeptidase activity of *Vc*Hsp31, measured at seven different temperatures (Fig 7d), indicate a maximum amidopeptidase activity at 37°C. Temperature mediated opening up of the catalytic pocket possibly renders Cys188 more accessible to the substrates accounting for its increased peptidase activity. To further establish it two Phe residues Phe30 and Phe211, residing at the mouth/opening of the cavity, were mutated to Leu and the amidopeptidase activity of the resulting double mutant was measured. Amino acids corresponding to these Phe residues were previously proposed to play a key role to conquer the inaccessibility of putative catalytic triad in case of *Ec*Hsp31 [15]. Phe30 and Phe211 were mutated to Leu to partially retain the hydrophobic packing contribution of these side chains, if any, towards the overall structure. The double mutant exhibits nearly double amidopeptidase activity than the wild type (Fig 8b). Kinetic characterization revealed the Michaelis constant (K_m_), catalytic constant k_cat_ and specificity constant (k_cat_/K_m_) of the amidopeptidase activity of VcHsp31 are 112.5 μM, 0.0765 min^-1^ and 0.681 mM^-1^min^-1^. There are number of residues around the vicinity of active site those are highly conserved in Class-I Hsp31 homologs (Fig 8a). Alanine mutant of these residues indicate that mutant C188A showed severe reduction in amidopeptidase activity followed by the H189A, further confirming the role of these two catalytic residues to Hsp31 family (Fig 8a and 8b). The reduced amidopeptidase activity of H158A could be attributed as the partial loss of the ‘oxyanion-hole’ which is required to stabilize the transition state during proteolysis. Reduction of amidopeptidase activity of Y224A is even more than that of H158A. Three residues, Tyr224, Phe71 and Met225 are highly conserved in the Class-I Hsp31 and His158 which is part of the ‘oxyanion hole’ packs snugly around these residues. We believe that mutation of any of these residues would increase the conformational freedom of His158 side chain and disturb the ‘oxyanion hole’ sub-structure. Tyr224 might also serve as a sub-site to bind the substrate and therefore its mutation is reducing the amidopeptidase activity. E79A, on the other hand has emerged as a gain-of-function mutant with enhanced amidopeptidase activity (Fig 8b). Divalent cations like Mg^2+^ and Ca^2+^ have nominal effect on the amidopeptidase activity of *Vc*Hsp31 while its activity is almost abolished (>90%) in the presence of Zn^2+^, Pb^2+^ and Hg^2+^ (S3 Fig).

### VcHsp31 has methyl glyoxalase activity

The Michaelis constant (K_m_), V_max_, catalytic constant (K_cat_) and specificity constant (K_cat_/K_m_) of the glutathione independent methylglyoxalase activity calculated of the wild type protein is 11.932 μM min^-1^, 0.25084 mM, 39.77 min^-1^ and 0.15854 M^-1^ min^-1^, respectively (Fig 8c). Glyoxalase activity is also measured for different mutants and compared with the wild type. Two among three catalytic triad mutants: C188A, H189A and residues present in the vicinity of the active site such as H158A, Y224A, H76A and E79A were tested for this purpose and the resulting data is shown in Fig 8d. Only H158A has retained about half of the wild type activity (~53%) while all the other mutants have almost immeasurable activity.

### VcHsp31 has chaperone activity

**Fig 9:**
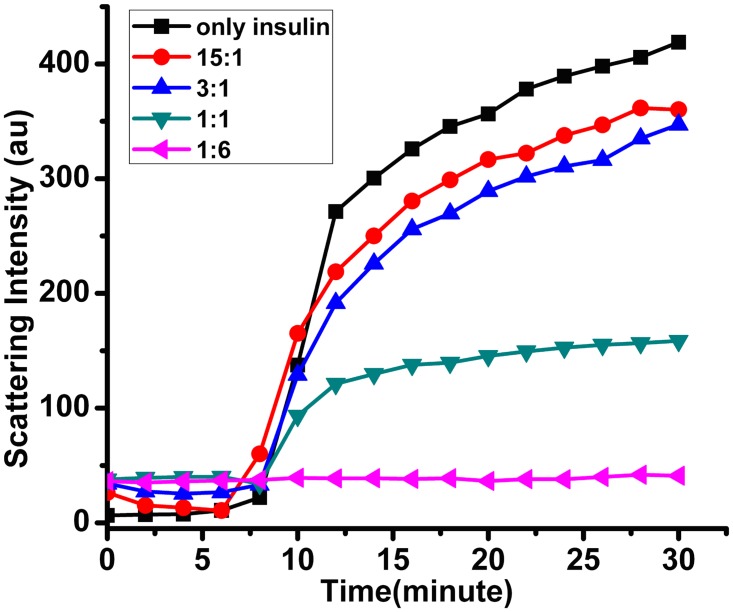
*Vc*Hsp31 inhibits in vitro protein aggregation. DTT induced aggregation of insulin and suppression by VcHsp31 at 35°C. Insulin (stock concentration 3 mg/ml in 20 mM Tris buffer, pH-8.0, containing 150 mM NaCl dissolved in presence of 20 mM NaOH) was reduced with 25 mM DTT in a final volume of 1.2 ml and aggregation of insulin in the absence of VcHsp31(black trace) and presence of VcHsp31 with insulin in the substrate:enzyme ratio of 15:1 (red trace), 3:1 (blue trace), 1:1 (green trace) and 1:6 (violet trace) was monitored by measuring the right angle light scattering at 360 nm. The graph is a representative plot (plotted in originpro 8) of three individual experiments.

The *hcha* gene product of *V*. *cholerae* O395 belongs to DJ-1/PfpI superfamily and annotated as a hypothetical intracellular protease/amidase. However, direct functional studies demonstrating chaperone activity have not been conducted on this protein to support its designation within the Hsp family. The influence of *Vc*Hsp31 on the aggregation of substrate proteins was measured using the model substrates insulin, which was widely used as substrate to demonstrate the ability to suppress aggregation in case of DJ-1 and recently in case of *Ec*Hsp31. DTT induced reduction of disulphide bond leads to unfolding and aggregation of insulin. The aggregation reaction can be monitored by simple right angle light scattering. Fig 9 shows the insulin aggregation kinetics due to light scattering at 360 nm. Aggregation starts after 7–8 minutes of DTT addition and keeps increasing before it reaches a saturation level by 20–25 minutes. Interestingly this aggregation can be suppressed by VcHsp31 in a concentration dependent manner. *Vc*Hsp31 suppress insulin aggregation, induced by reducing agent, efficiently and in a dose dependent manner establishing its role in chaperone activity.

## Discussion

Heat shock proteins (Hsps) are molecular chaperones which upon exposure to environmental stresses like elevated temperature play an obligatory role to maintain the properly folded protein pool in the cells. It has been reported that exposure of *E*. *coli* cells to 50°C causes induction of 77 genes and most of them are transcribed into Hsps [43]. Our knowledge about Class-I Hsp31 relies on the structural [6,44] and functional [16,19] characterization of homodimeric *Ec*Hsp31 [6]. Each monomer of *Ec*Hsp31consists of, A-domain and P-domain, connected by a flexible linker [6]. Dimerization through P-domains creates a hydrophobic bowl shaped structure capable to capture early unfolded intermediates [6]. Movement of the linker region at high temperature was proposed to uncover the hydrophobic surface accounting for its higher chaperone activity [16].

Crystal structure of *Vc*Hsp31, reported here, shows unambiguous electron density for the flexible linker, irrespective of at what temperature the crystals were grown (Fig 3c). This is interesting because the linker loop and its neighbouring loops are not involved in crystal packing, indicating that the loop is not as flexible as *Ec*Hsp31. The intriguing question is how *Vc*Hsp31 exhibit chaperone activity at elevated temperature? Occurrence of two distinct types of dimers, Type-I and Type-II, provide a reason behind this. At elevated temperature, the percentage of Type-II dimer increases with associated opening of hydrophobic patches (Fig 5e) which was otherwise buried in Type-I dimer (Fig 5d). This can be attributed to a temperature mediated dissociation of dimeric interface to accommodate the unfolded polypeptide chains at high thermal shock. Interestingly, the hydrophobic surface which is exposed upon swinging is close to the hydrophobic ‘bowl region’ and therefore expands the existing hydrophobic patch at the bowl. At lower temperature (e.g., 18°C), as observed in case of E79A-*Vc*Hsp31, all dimers are in Type-I form which possess minimal chaperone activities. With different stresses such as high pH and temperature, Type-I dimers are transformed into Type-II dimers to account the cellular requisites. With various structural transitions such as loosing of critical ionic interactions at the dimeric interface and exposing of various hydrophobic residues, activated Type-II dimers are predominate in hostile situation. This can be correlated well with the fact that pathogenic *V*. *cholerae* colonize in human small intestine where the environment is in higher pH and temperature from its non-colonizing state. At this new environment, many proteins get misfolded or inactivated rapidly and *Vc*Hsp31 can act as a saviour for *V*. *cholerae*. At stressed conditions, unfolded polypeptide chains either can wedge at the surface of *Vc*Hsp31 to get released when condition is favourable or are degraded into individual amino acids. The highly negative waist region of the dimers may predominantly bind polypeptides comprising of mostly positively charged residues which is in accordance with the fact that a typical chaperone target is made of hydrophobic amino acids flanked by basic residues [7]. One may argue that the different dimers seen here may be due to packing artefact. The unit cell of *Vc*Hsp31^20C^ and *Vc*Hsp31^25C^ is same but different from C188A-*Vc*Hsp31, yet all of them contain two types of dimers. On the other hand, mutants E79A-*Vc*Hsp31 and C188A-*Vc*Hsp31 have the same cell but the former exclusively contains Type-I dimer while the later contains both Type-I and Type-II forms. These two facts rule out the possibility of packing artefact. Moreover, since the mutations were located deep in the catalytic pocket and not on the surface, possibility of packing difference due to the mutations can also be ruled out.

Despite the presence of ‘Cys-His-Asp’ catalytic triad and an oxyanion hole (made of His158 and Gly156, Gly157) close to the SG of Cys188, *Vc*Hsp31 (or its homologs) cannot be qualified as a good proteolytic enzyme even at optimum pH (8.0) and temperature (37°C). Temperature dependent unlocking of buried active site along with several structural transformations is prerequisite for its optimal functioning. Shielding of Cys188 attributes to its lower functional state and therefore, partial removal of the shielding through F30L/F211L mutations nearly doubles the peptidase activity (Fig 8b). The size and shape of the cavities, through which the catalytic Cys is accessed, is also different between *Vc*Hsp31 and *Ec*Hsp31 which may imply their different substrate specificities. Severe reduction of activity in case of C188A, followed by H189A, further confirms their roles as catalytic residues in Hsp31 family (Fig 8a and 8b). Reduced activity of H158A could be attributed to the partial loss of the ‘oxyanion-hole’ despite the fact that Tyr224, Phe71 and Met225 stabilize the ‘oxyanion hole’ sub-structure. E79A, on the other hand, has emerged as a gain-of-function mutant with enhanced amidopeptidase activity (Fig 8b). Apart from the catalytic triad, this glutamate residue is highly conserved in all three orthologs of Hsp31 including DJ-1 family [45]. At our working pH (> 5.0) Glu79 is negatively charged and it may interact with sulfhydryl hydrogen of Cys188, outcompeting the interactions between Cys188 and His189. As a result, a considerable population of Cys188 side-chain conformation is engaged to interact with Glu79 rather than interacting with His189, rendering Cys188 less neucleophile than it would have been in the absence of Glu79. Therefore, the presence of this conserved glutamate might be attributed to the attenuation of the amidopeptidase function in the Hsp31 orthologs.

Furthermore, glutathione independent methylglyoxalase activity of *Vc*Hsp31 and its mutants has little effect on the cavity size. At any circumstance, glyoxalates are highly toxic electrophiles for cellular system which covalently modify proteins, nucleic acids and so on [46]. Removal of glyoxalates from metabolic pool is prerequisite for maintaining the cellular homeostasis. Glutathione independent glyoxalase activity has been recently reported for *Ec*Hsp31 [25]. Cys188, His189, His76 and Glu79 are the four most important residues for glyoxalase activity of *Vc*Hsp31. Mutations of any of these residues to alanine yielded almost 10 folds lower glyoxalase activity compared to the wild type (Fig 8d). Similar to reduced glutathione, the thiol group of Cys188 can act as an electrophile, where hemithioacetal is formed before the rate limiting enzyme catalyzing step. As cytosolic space of Gram-negative bacterium like *V*. *cholerae* is in highly reduced state, glyoxalase activity could dominate in *Vc*Hsp31 than the other functional properties to control the protein modification. Additionally, while Tyr224 acts as a substrate recognition subsite, His76 and His158 further stabilize the oxyanion hole, since mutations of these residues to alanine yielded significantly reduced glyoxalase activity of *Vc*Hsp31 (Fig 8d).

With all these repertoire of activities, *Vc*Hsp31 has emerged as an important chaperone-protease with novel inter-dimeric motion to control protein homeostasis in stressed condition along with glyoxalase activity to monitor the protein modification level. Poor accessibility of the catalytic triad, together with the strategic location of Glu79, significantly weakens the amidopeptidase activity of *Vc*Hsp31 which in turn facilitates to control the cellular level of proteolysis. In contrast, location of Glu79 is beneficial for proton transfer during methyl glyoxalase activity. Recent findings [24], along with our current report indicating the glyoxalase activity for *Vc*Hsp31, could contribute to a pivotal role in controlling the protein ensembles and carbohydrate modification. As similar residues are involved both in amidopeptidase and glyoxalase activities, *Vc*Hsp31 can switch from one functional state to the other depending on the cellular constraint. Moreover, from the crystal structures of *Vc*Hsp31 we described two distinct types of dimers, resulting through temperature induced swinging motion of the monomers, which differ by a sizeable amount of hydrophobic patch near the bowl. This newly unburied patch could function in coherence to the existing hydrophobic patch at the bowl and provide another potential mechanism to bind unfolded substrate at high temperature. Finally, *Vc*Hsp31, a second representative of Class-I Hsp31s have come into sight by exhibiting unique swinging motion for chaperone activity and ensembles of functional properties.

## Supporting information

S1 Fig Existence of dimeric VcHsp31 in solution. (a) FPLC analysis and elution profile of *Vc*Hsp31 ran in a Superdex-200 increase column (GE Healthcare). The elution volume of the column corresponds to a dimeric VcHsp31 species. (b) Standard curve [Ve/V0 (elution volume/void volume) vs. log of protein molecular weight (MW in kDa)] is drawn based on the elution volumes of protein mixtures of known molecular weight (Albumin 66.5 kDa; Ovalbumin 45 kDa; Chymotrypsin 25 kDa and Ribonuclease A 13.7 kDa). VcHsp31 is shown in red symbol.
(DOCX)

S2 Fig Protease and peptidase activity of VcHsp31. (a) 15% SDS-PAGE showing the protease activity of *Vc*Hsp31 on BSA at pH 6.5, 7.2 and 8.0 at 20°C. The reactions were monitored for 40 hours. Around 40% cleavage of BSA has been observed after 40 hrs of incubation at 20°C (pH 8.0) (Top). Better protease activity has been found at 37°C as complete cleavage of BSA has been observed within 24 hrs (Bottom); (b) Amidopeptidase activity at 37°C with time; (c) Peptidase activity of VcHsp31 with Ala-AMC was assayed at 37°C at pH-8.0 upto 7 hours. From the substrate saturation curve the michaelis constant, catalytic constant, specificity constant of wild type VcHsp31 was measured as 112.5 μM, 0.0765 min^-1^ and 0.681 mM^-1^min^-1^ respectively. Each rate was measured in triplicate, and mean values are plotted in originpro8 with standard deviations (calculated in Microsoft excel) shown as error bars.
(DOCX)

S3 Fig Role of metal ions in Vchsp31 structure and function. (a) Effects of different metal ions on the peptidase activity of *Vc*Hsp31. (b) 2-His-1-Caboxylate motif of *Ec*Hsp31 (yellow) that binds a Zn^2+^. In *Vc*Hsp31 (green) this motif is replaced by Tyr125-Asp88-Ser93 which is unable to bind Zn^2+^.
(DOCX)

## References

[1] Yura T, Nagai H, Mori H. Regulation of the heat-shock response in bacteria. Annu Rev Microbiol. 1993;47: 321–350. 10.1146/annurev.mi.47.100193.001541 7504905

[2] Hartl FU, Hayer-Hartl M. Molecular chaperones in the cytosol: from nascent chain to folded protein. Science. 2002;295: 1852–1858. 10.1126/science.1068408 11884745

[3] Lee S, Tsai FT. Molecular chaperones in protein quality control. J Biochem Mol Biol. 2005;38: 259–265. 15943899 10.5483/bmbrep.2005.38.3.259

[4] Wu C. Heat shock transcription factors: structure and regulation. Annu Rev Cell Dev Biol. 1995;11: 441–469. 10.1146/annurev.cb.11.110195.002301 8689565

[5] Gottesman S, Wickner S, Maurizi MR. Protein quality control: triage by chaperones and proteases. Genes Dev. 1997;11: 815–823. 9106654 10.1101/gad.11.7.815

[6] Quigley PM, Korotkov K, Baneyx F, Hol WG. The 1.6-A crystal structure of the class of chaperones represented by Escherichia coli Hsp31 reveals a putative catalytic triad. Proc Natl Acad Sci U S A. 2003;100: 3137–3142. 10.1073/pnas.0530312100 12621151 PMC152259

[7] Sharma SK, Christen P, Goloubinoff P. Disaggregating chaperones: an unfolding story. Curr Protein Pept Sci. 2009;10: 432–446. 19538153 10.2174/138920309789351930

[8] Graille M, Quevillon-Cheruel S, Leulliot N, Zhou CZ, Li de la Sierra Gallay I, Jacquamet L, et al Crystal structure of the YDR533c S. cerevisiae protein, a class II member of the Hsp31 family. Structure. 2004;12: 839–847. 10.1016/j.str.2004.02.030 15130476

[9] Lee SJ, Kim SJ, Kim IK, Ko J, Jeong CS, Kim GH, et al Crystal structures of human DJ-1 and Escherichia coli Hsp31, which share an evolutionarily conserved domain. J Biol Chem. 2003;278: 44552–44559. 10.1074/jbc.M304517200 12939276

[10] Bandyopadhyay S, Cookson MR. Evolutionary and functional relationships within the DJ1 superfamily. BMC Evol Biol. 2004;4: 6 10.1186/1471-2148-4-6 15070401 PMC385224

[11] Lucas JI, Marin I. A new evolutionary paradigm for the Parkinson disease gene DJ-1. Mol Biol Evol. 2007;24: 551–561. 10.1093/molbev/msl186 17138626

[12] Wei Y, Ringe D, Wilson MA, Ondrechen MJ. Identification of functional subclasses in the DJ-1 superfamily proteins. PLoS Comput Biol. 2007;3: e10 10.1371/journal.pcbi.0030010 17257049 PMC1782040

[13] Parsot C, Mekalanos JJ. Expression of ToxR, the transcriptional activator of the virulence factors in Vibrio cholerae, is modulated by the heat shock response. Proc Natl Acad Sci U S A. 1990;87: 9898–9902. 2124707 10.1073/pnas.87.24.9898 PMC55281

[14] Das S, Dey S, Roy T, Sen U. Cloning, expression, purification, crystallization and preliminary X-ray analysis of the 31 kDa Vibrio cholerae heat-shock protein VcHsp31. Acta Crystallogr Sect F Struct Biol Cryst Commun. 2011;67: 1382–1385. 10.1107/S1744309111032970 22102237 PMC3212456

[15] Quigley PM, Korotkov K, Baneyx F, Hol WG. A new native EcHsp31 structure suggests a key role of structural flexibility for chaperone function. Protein Sci. 2004;13: 269–277. 10.1110/ps.03399604 14691241 PMC2286521

[16] Sastry MS, Quigley PM, Hol WG, Baneyx F. The linker-loop region of Escherichia coli chaperone Hsp31 functions as a gate that modulates high-affinity substrate binding at elevated temperatures. Proc Natl Acad Sci U S A. 2004;101: 8587–8592. 10.1073/pnas.0403033101 15173574 PMC423238

[17] Choi D, Ryu KS, Park C. Structural alteration of Escherichia coli Hsp31 by thermal unfolding increases chaperone activity. Biochim Biophys Acta. 2013;1834: 621–628. 10.1016/j.bbapap.2012.11.006 23202248

[18] Sastry MS, Zhou W, Baneyx F. Integrity of N- and C-termini is important for E. coli Hsp31 chaperone activity. Protein Sci. 2009;18: 1439–1447. 10.1002/pro.158 19517531 PMC2775212

[19] Sastry MS, Korotkov K, Brodsky Y, Baneyx F. Hsp31, the Escherichia coli yedU gene product, is a molecular chaperone whose activity is inhibited by ATP at high temperatures. J Biol Chem. 2002;277: 46026–46034. 10.1074/jbc.M205800200 12235139

[20] Malki A, Caldas T, Abdallah J, Kern R, Eckey V, Kim SJ, et al Peptidase activity of the Escherichia coli Hsp31 chaperone. J Biol Chem. 2005;280: 14420–14426. 10.1074/jbc.M408296200 15550391

[21] Cooper RA, Anderson A. The formation and catabolism of methylglyoxal during glycolysis in Escherichia coli. FEBS Lett. 1970;11: 273–276. 11945504 10.1016/0014-5793(70)80546-4

[22] Bucala R, Cerami A. Advanced glycosylation: chemistry, biology, and implications for diabetes and aging. Adv Pharmacol. 1992;23: 1–34. 1540533 10.1016/s1054-3589(08)60961-8

[23] Ahmed MU, Thorpe SR, Baynes JW. Identification of N epsilon-carboxymethyllysine as a degradation product of fructoselysine in glycated protein. J Biol Chem. 1986;261: 4889–4894. 3082871

[24] Mihoub M, Abdallah J, Gontero B, Dairou J, Richarme G. The DJ-1 superfamily member Hsp31 repairs proteins from glycation by methylglyoxal and glyoxal. Biochem Biophys Res Commun. 2015;463: 1305–1310. 10.1016/j.bbrc.2015.06.111 26102038

[25] Subedi KP, Choi D, Kim I, Min B, Park C. Hsp31 of Escherichia coli K-12 is glyoxalase III. Mol Microbiol. 2011;81: 926–936. 10.1111/j.1365-2958.2011.07736.x 21696459

[26] Bankapalli K, Saladi S, Awadia SS, Goswami A V, Samaddar M, D’Silva P. Robust glyoxalase activity of Hsp31, a ThiJ/DJ-1/PfpI family member protein, is critical for oxidative stress resistance in Saccharomyces cerevisiae. J Biol Chem. 2015;290: 26491–26507. 10.1074/jbc.M115.673624 26370081 PMC4646309

[27] McCoy AJ, Grosse-Kunstleve RW, Adams PD, Winn MD, Storoni LC, Read RJ. Phaser crystallographic software. J Appl Crystallogr. 2007;40: 658–674. 10.1107/S0021889807021206 19461840 PMC2483472

[28] Adams PD, Afonine P V, Bunkoczi G, Chen VB, Davis IW, Echols N, et al PHENIX: a comprehensive Python-based system for macromolecular structure solution. Acta Crystallogr D Biol Crystallogr. 2010;66: 213–221. 10.1107/S0907444909052925 20124702 PMC2815670

[29] Winn MD, Ballard CC, Cowtan KD, Dodson EJ, Emsley P, Evans PR, et al Overview of the CCP4 suite and current developments. Acta Crystallogr D Biol Crystallogr. 2011;67: 235–242. 10.1107/S0907444910045749 21460441 PMC3069738

[30] Emsley P, Cowtan K. Coot: model-building tools for molecular graphics. Acta Crystallogr D Biol Crystallogr. 2004;60: 2126–2132. 10.1107/S0907444904019158 15572765

[31] Krissinel E, Henrick K. Inference of macromolecular assemblies from crystalline state. J Mol Biol. 2007;372: 774–797. 10.1016/j.jmb.2007.05.022 17681537

[32] Thompson JD, Higgins DG, Gibson TJ. CLUSTAL W: improving the sensitivity of progressive multiple sequence alignment through sequence weighting, position-specific gap penalties and weight matrix choice. Nucleic Acids Res. 1994;22: 4673–4680. 7984417 10.1093/nar/22.22.4673 PMC308517

[33] Do CB, Katoh K. Protein multiple sequence alignment. Methods Mol Biol. 2008;484: 379–413. 10.1007/978-1-59745-398-1_25 18592193

[34] Schuyler AD, Chirikjian GS. Normal mode analysis of proteins: a comparison of rigid cluster modes with C(alpha) coarse graining. J Mol Graph Model. 2004;22: 183–193. 10.1016/S1093-3263(03)00158-X 14629977

[35] Gilbert RP, Brandt RB. Spectrophotometric determination of methyl glyoxal with 2,4-dinitrophenylhydrazine. Anal Chem. 1975;47: 2418–2422. 1190480 10.1021/ac60364a003

[36] Kwon K, Choi D, Hyun JK, Jung HS, Baek K, Park C. Novel glyoxalases from Arabidopsis thaliana. Febs J. 2013;280: 3328–3339. 10.1111/febs.12321 23651081

[37] Tsai CJ, Aslam K, Drendel HM, Asiago JM, Goode KM, Paul LN, et al Hsp31 Is a Stress Response Chaperone That Intervenes in the Protein Misfolding Process. J Biol Chem. 2015;290: 24816–24834. 10.1074/jbc.M115.678367 26306045 PMC4598993

[38] Laskowski RA, MacArthur MW, Thornton JM. Validation of protein models derived from experiment. Curr Opin Struct Biol. 1998;8: 631–639. 9818269 10.1016/s0959-440x(98)80156-5

[39] Zhan D, Bai A, Yu L, Han W, Feng Y. Characterization of the PH1704 protease from Pyrococcus horikoshii OT3 and the critical functions of Tyr120. PLoS One. 2014;9: e103902 10.1371/journal.pone.0103902 25192005 PMC4156298

[40] Du X, Choi IG, Kim R, Wang W, Jancarik J, Yokota H, et al Crystal structure of an intracellular protease from Pyrococcus horikoshii at 2-A resolution. Proc Natl Acad Sci U S A. 2000;97: 14079–14084. 10.1073/pnas.260503597 11114201 PMC18874

[41] Wilson MA, St Amour C V, Collins JL, Ringe D, Petsko GA. The 1.8-A resolution crystal structure of YDR533Cp from Saccharomyces cerevisiae: a member of the DJ-1/ThiJ/PfpI superfamily. Proc Natl Acad Sci U S A. 2004;101: 1531–1536. 10.1073/pnas.0308089100 14745011 PMC341769

[42] Ollis DL, Cheah E, Cygler M, Dijkstra B, Frolow F, Franken SM, et al The alpha/beta hydrolase fold. Protein Eng. 1992;5: 197–211. 1409539 10.1093/protein/5.3.197

[43] Richmond CS, Glasner JD, Mau R, Jin H, Blattner FR. Genome-wide expression profiling in Escherichia coli K-12. Nucleic Acids Res. 1999;27: 3821–3835. 10481021 10.1093/nar/27.19.3821 PMC148645

[44] Zhao Y, Liu D, Kaluarachchi WD, Bellamy HD, White MA, Fox RO. The crystal structure of Escherichia coli heat shock protein YedU reveals three potential catalytic active sites. Protein Sci. 2003;12: 2303–2311. 10.1110/ps.03121403 14500888 PMC2366919

[45] Li H, Ryan TJ, Chave KJ, Van Roey P. Three-dimensional structure of human gamma -glutamyl hydrolase. A class I glatamine amidotransferase adapted for a complex substate. J Biol Chem. 2002;277: 24522–24529. 10.1074/jbc.M202020200 11953431

[46] Hasim S, Hussin NA, Alomar F, Bidasee KR, Nickerson KW, Wilson MA. A glutathione-independent glyoxalase of the DJ-1 superfamily plays an important role in managing metabolically generated methylglyoxal in Candida albicans. J Biol Chem. 2014;289: 1662–1674. 10.1074/jbc.M113.505784 24302734 PMC3894345

