# Supplementary material for: Structural and biochemical studies on Vibrio cholerae Hsp31 reveals a novel dimeric form and Glutathione-independent Glyoxalase activity
Source: PLoS One. 2017 Feb 24;12(2):e0172629. doi: 10.1371/journal.pone.0172629 (PMC5325305; doi:10.1371/journal.pone.0172629)
Supplement: S1 Fig — (a) FPLC analysis and elution profile of VcHsp31 ran in a Superdex-200 increase column (GE Healthcare). The elution volume of the column corresponds to a dimeric VcHsp31 species. (b) Standard curve [Ve/V0 (elution volume/void volume) vs. log of protein molecular weight (MW in kDa)] is drawn based on the elution volumes of protein mixtures of known molecular weight (Albumin 66.5 kDa; Ovalbumin 45 kDa; Chymotrypsin 25 kDa and Ribonuclease A 13.7 kDa). VcHsp31 is shown in red symbol. (DOCX) [file pone.0172629.s001.docx]

**Supporting Figure S1**


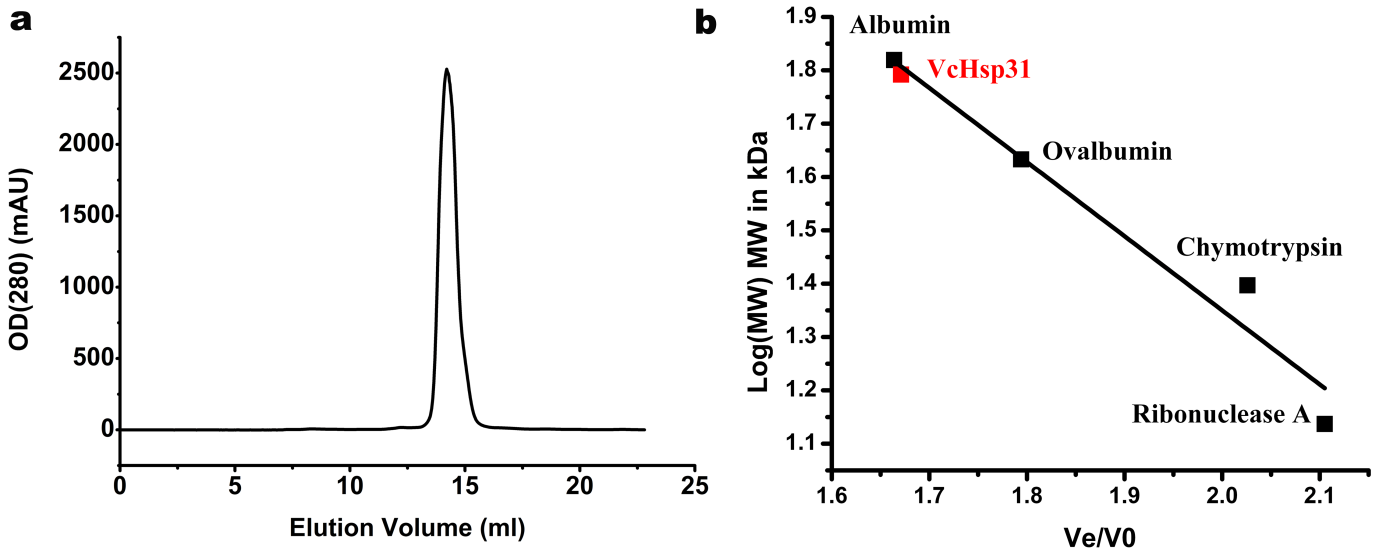


**S1 Fig**: **Existence of dimeric VcHsp31 in solution**. (a) FPLC analysis and elution profile of *Vc*Hsp31 ran in a Superdex-200 increase column (GE Healthcare). The elution volume of the column corresponds to a dimeric VcHsp31 species. (b) Standard curve [Ve/V0 (elution volume/void volume) vs. log of protein molecular weight (MW in kDa)] is drawn based on the elution volumes of protein mixtures of known molecular weight (Albumin 66.5 kDa; Ovalbumin 45 kDa; Chymotrypsin 25 kDa and Ribonuclease A 13.7 kDa). VcHsp31 is shown in red symbol.
