# Supplementary material for: Structural and biochemical studies on Vibrio cholerae Hsp31 reveals a novel dimeric form and Glutathione-independent Glyoxalase activity
Source: PLoS One. 2017 Feb 24;12(2):e0172629. doi: 10.1371/journal.pone.0172629 (PMC5325305; doi:10.1371/journal.pone.0172629)
Supplement: S2 Fig — (a) 15% SDS-PAGE showing the protease activity of VcHsp31 on BSA at pH 6.5, 7.2 and 8.0 at 20°C. The reactions were monitored for 40 hours. Around 40% cleavage of BSA has been observed after 40 hrs of incubation at 20°C (pH 8.0) (Top). Better protease activity has been found at 37°C as complete cleavage of BSA has been observed within 24 hrs (Bottom); (b) Amidopeptidase activity at 37°C with time; (c) Peptidase activity of VcHsp31 with Ala-AMC was assayed at 37°C at pH-8.0 upto 7 hours. From the substrate saturation curve the michaelis constant, catalytic constant, specificity constant of wild type VcHsp31 was measured as 112.5 μM, 0.0765 min-1 and 0.681 mM-1min-1 respectively. Each rate was measured in triplicate, and mean values are plotted in originpro8 with standard deviations (calculated in Microsoft excel) shown as error bars. (DOCX) [file pone.0172629.s002.docx]

**Supporting Figure S2**

**
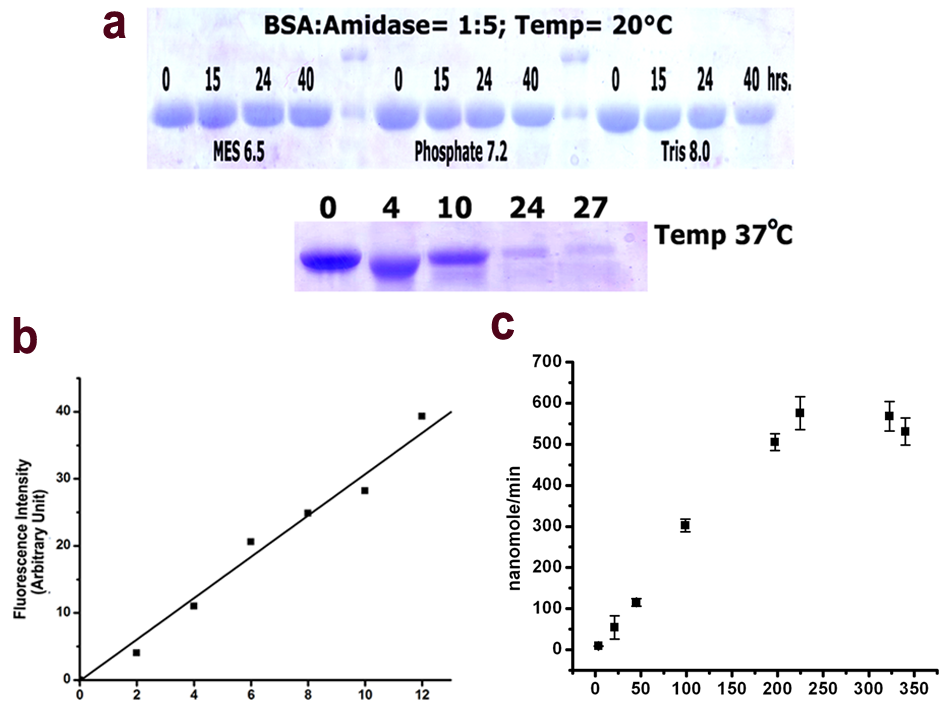
**

**S2 Fig**: **Protease and peptidase activity of VcHsp31.** (a) 15% SDS-PAGE showing the protease activity of *Vc*Hsp31 on BSA at pH 6.5, 7.2 and 8.0 at 20°C. The reactions were monitored for 40 hours. Around 40% cleavage of BSA has been observed after 40 hrs of incubation at 20°C (pH 8.0) (Top). Better protease activity has been found at 37°C as complete cleavage of BSA has been observed within 24 hrs (Bottom); (b) Amidopeptidase activity at 37°C with time; (c) Peptidase activity of VcHsp31 with Ala-AMC was assayed at 37°C at pH-8.0 upto 7 hours. From the substrate saturation curve the michaelis constant, catalytic constant , specificity constant of wild type VcHsp31 was measured as 112.5 µM, 0.0765 min^-1^ and 0.681 mM^-1^min^-1^ respectively. Each rate was measured in triplicate, and mean values are plotted in originpro8 with standard deviations (calculated in Microsoft excel) shown as error bars.
